# Monthly Summary

**Published:** 1868-11

**Authors:** 


### MONTHLY SUMMARY.

*Nerves of the Neurilemma.*—At a recent meeting of the Academy of Sciences, Paris, Prof. Robin presented an important communication from M. Sappey. M. Sappey's memoir bears on the, discovery of certain nervous filaments which have the same relations towards the nerves as have the vasa vasorum towards the vessels. But, through the novelty and great interest of this paper, we believe it well worth while to translate a few of its principal passages.

"The neurilemma receives nervous filaments, which are in the same relation towards the nerves as the vasa vasorum towards the vessels; I therefore propose that they should be designated by the name of *nervi nervorum.* Their existence in the fibrous sheath of nerves had not been signalized; yet it is undeniable and may be easily demonstrated.

The disposition which the *nervi nervornm* effect in the neurilemma differs in but a slight degree from the one presented by the nervous ramifications in the other dependencies of the fibrous system. Like these, they generally attend the arteries; in like manner they exchange, during their course numerous divisions, by weich anastomoses are effected, so that on different points of their situation a small plexus may be observed, showing irregular and unequal meshes.

It ie nor only on the principal or common shearh that they are met with, but likewise on those which involve the principal fasciculi and the tertiary fasciculi. I have been able to trace them even on the sheath of secondary fasciculi. But in proportion as the size of the sheath diminishes, they become far more minute, and are much seldomer met with. They are never seen to extend to the involving membrahe of primitive fasciculi.

The absence of the *nervi nervorum* on the sheath of primitive fasciculi explains why they are not met with on all uervous divisions the diameter of which does not exceed one millimetre.

The lining or profound membrane of the optic nerve, which fulfils the office of a neurilemma, does not receive any nervous filament. The exterioa membrane, on the contrary, receives a great number of fflaments, which take their origin in the ciliary nerves.

The outward sheath of the optic nerves, so rich in *nervi nervorum*, is remarkabie also for the abundance of elastic fibres which enter into its composition. The ancients were therefore thoroughly mistaken in censidering it a sort of link between the dura mater and the sclerotica, or, in other words, as prolonging the one and being prolonged by the other. It differs widely from the two structures—first, by its elastic fibres, which are wanting in both ; and, secondly, by its *nervi nervorum*, which are extremely rare in the dura mater of the brain, and of wbich there exists no vestige in the sclerotic. Consequently, anatomical analysis. far from confirming the analogy which had been pointed out by so great a number of anatomists, attests, on the contrary, that it differs by its own proper characteristics from the other two membranes, with which it is continuous."—*Paris Correspondence of the London Lancet.*

---

*How to Utilize Leeches.*—The German doctors have lately been playing their leeches a droll trick—making one worm do the work of many. When the little blood-sucker has taken his fill and is about to release his bite, he is tapped ; a small incision is made in his side, that serves as an outlet for the blood, and he goes on sucking, in happy ignorance of the cause of his abnormal appetite, as long as the doctor pleases. Bdellatomy is the name given to the practice, and it is urged that it is not cruel, but contrarywise, since it does the leech a good turn by enabling him to enjoy his rich feast indefinitely. He does not die under the operation, but with proper treatment is soon healed, and may be incised over and over again. There was once an alderman who wished he had been a camel, that he might have been blessed with the seven stomachs vouchsafed by nature to that animal. If such a gormand still exists, let him seek surgical aid in some such treatment as that practiced on the leeches, that he may eat and drink *ad libitum*, and feel no worse.—*Once a Week.*

*Imprudent Application of Carbolic Acid.*—By Prof. P. H· Vander Weyde, M. D. of N. Y. Mr. Berger, a London chemist, lately killed himself by carbolic acid, in attempting to introduce a drop of it into a tooth for the cure of the tooth ache. The London Journals say that the remedy was a new one, and that he died as a martyr of science by attempting to test this new remedy ; but when we take into consideration that creasotc has been used for years for this same purpose, and that this substance owes its principle properties to the carbolic acid it contains, (it being in fact only impure carbolic acid) there is nothing new in the remedy, besides some dentists in this country have substituted for the creasote, and I have in the last two or three years prescribed it exclusively for the same purpose. The way to use it is to place a drop on a small cotton plug, and introduce it into the cavity, it is a way which will suggest itself to any one of common sense, even one who has never seen dental operations· Mr. Berger, however, acted in a most foolish, stupid, and imprudent manner. Dr. Metcalf testifies that he (Berger) had fixed an elastic tube 10 feet long to a large glass jar with carbolic acid, had then seated himself in a chair, and inserted the end of the tube in his mouth, for the purpose of allowing a drop of the liquid to fall into his tooth. He had a brass regulator on the tube to control the quantity of the acid, but it did not act right, and the volatile poison overcame him, he became giddy and fell. Being alone in the room the poison continued flowing, the heart's action was stopped and he died. Comment is unnecessary.

---

*Extensive Removal of Bones of the Face.*—In the *Galveston* Med. Journal, Dr. J. D. Rankin of Palestine Texas, reports the extirpation of the entire half of the superior maxillary bone, the whole of the malar bone, the entire orbital plate of the superior maxillary bone, the orbital plate of the ethmoid bone, and the external angular process of the frontal bone forming the superior portion of the eye," involving of course the removal of the remains of the globe of the eye which had previously ruptured. The operation was for the relief of a necrosis, consequent to suppuration excited by a diseased tooth. He says " there was not a great deal of blood lost, and but little fever following it." The paient made a rapid recovery.

*Remarkable Dental Operations*—The New York correspondent of the *Continental Gazette* of Paris, of May 16th, mentions two remarkable operations in oral surgery in which the advantage of the dental art was conspicuously displayed :

"The first was the dissecting of the lower jaw and half the upper, the supplying of new jaws of vulcanite rubber, a new palatine arch, and artificial palate. The operation was performed by Dr. J. M. CARNOCHAN, and the artificial replacement by Dr. G. H. PERRINE. I have seen a letter from the patient, written at Oregon City, and he denominates the case a perfect success, after having an experience of several months; he cannot sufficiently express his gratitude. In the second case there is amputation of the lower jaw, and replacement in rubber, and it involves the loss of three-fourths of the tongue, totally destroying articulation. Dr. PERRINE has this case, and undertakes to supply an artificial tongue which shall articulate distinctly, besides accomplishing the remainder of the restoration. These are the most remarkable cases, on record and deserve to be widely noticed. Dr. Perrine is a young man, enthusiastic in his profession, and remarkably successful."

"The last case has, I learn since, been complete in restoring speech and the contour of the features. Greater interest is attached to the first mentioned case, from the fact, that the patient applied to the best practitioners in Paris; not receiving sufficient encouragement he came to New York for treatment. In view of all the facts these are most remarkable combined triumphs of surgery and dentistry." [See history of case in present No. page 345. EDS.]

---

*Artificial Digestion*—Dr. W. Marcet has published a pamphlet on preparing food for weak stomachs, which the London *Medical Times and Gazette* speaks of in terms of high commendation. Dr. Marcet has contrived a method by which the natural process of digestion is imitated, so that food may be taken in a partially digested condition, and thus more nutriment can be managed by a weak stomach than it could otherwise dispose of.

He took hydrochloric acid and some pepsine, added these along with water to a quantity of meat, allowing the whole to simmer over a water bath at about the temperature of the body. When the meat was sufficiently broken up it was strained, and the acid neutralized by carbonate of soda, when it was ascertained that the product was of a most agreeable character, easily digestible, and containing a vast deal more nourishment than common beef tea. The proportions he recommends are, 58 grains of hydrochloric acid, sp. gr. 1.1496, in a pint (20 oz.) of water, with 15 grains of Boudault's pepsine, and 81 grains of bicarbonate of soda to a pound of meat, (weighed raw,) the chemicals costing about seven pence. Where pepsine is unattainable, strips of calves' stomach answer very well; or we do not see why the rennet prepared from it, and used for curdling milk, should not be employed. The food thus prepared keeps well until neutralized, but not so well afterward. One point to be noticed is, that no metallic vessel should be used in the process lest the acid act upon it.

---

*Insecticides.*—A recent traveller, JAGER, in Sketches of Travels in Singapore, Malacca and Java, published in 1866, furnishes some testimony of much interest on this subject. He says that a tincture prepared by macerating one part of *Pyrethrum roseum* in four parts of diluted alcohol, and then thinned with ten parts of water and applied to the skin, gave perfect security against attacks of mosquitoes and other insects, enabling him to pass the night in an open boat on the rivers of Siam without any annoyance; and moistening the beard and hands with the same liquid would protect the hunter for at least twelve hours from the flies. On the Island of Luzon, a board six inches wide was fastend horizontally all around his house, and a track of the powder, several inches in width, laid along this board, proved an insurmountable barrier to the incursions of the countless myriads of ants, infesting the country.—*Med. and Surg. Rep.*

---

*To Test the Purity of Water*—A glass tube of about a yard in length, closed at the lower end by a cork, upon which rests a white dish of porcelain, is recommended for determining the purity of water, as the slightest color is seen against the white ground, and the different shades indicate different ingredients. A green tinge is produced by minute algæ; a white opacity often by fungoid growths, iron salts by a peculiar ochry color. The apparatus is termed the chromiometer.

